# Co‐Regulating Solvation Structure and Hydrogen Bond Network via Bio‐Inspired Additive for Highly Reversible Zinc Anode

**DOI:** 10.1002/advs.202404968

**Published:** 2024-07-21

**Authors:** Sida Zhang, Qianzhi Gou, Weigen Chen, Haoran Luo, Ruduan Yuan, Kaixin Wang, Kaida Hu, Ziyi Wang, Changding Wang, Ruiqi Liu, Zhixian Zhang, Yu Lei, Yujie Zheng, Lei Wang, Fu Wan, Baoyu Li, Meng Li

**Affiliations:** ^1^ State Key Laboratory of Power Transmission Equipment Technology School of Electrical Engineering Chongqing University Chongqing 400044 China; ^2^ National Innovation Center for Industry‐Education Integration of Energy Storage Technology Chongqing University Chongqing 400044 China; ^3^ MOE Key Laboratory of Low‐grade Energy Utilization Technologies and Systems CQU‐NUS Renewable Energy Materials & Devices Joint Laboratory School of Energy & Power Engineering Chongqing University Chongqing 400044 China; ^4^ School of Building Services Science and Engineering Xi'an University of Architecture and Technology Xi'an 710055 China; ^5^ State Key Laboratory of Radiation Medicine and Protection School for Radiological and Interdisciplinary Sciences (RAD‐X) and Collaborative Innovation Center of Radiation Medicine of Jiangsu Higher Education Institutions Soochow University Suzhou 215123 China; ^6^ School of Electrical and Electronic Engineering Chongqing University of Technology Chongqing 400054 China

**Keywords:** aqueous zinc‐ion batteries, electrode interface, electrolyte additive, zinc anode

## Abstract

The feasibility of aqueous zinc‐ion batteries for large‐scale energy storage is hindered by the inherent challenges of Zn anode. Drawing inspiration from cellular mechanisms governing metal ion and nutrient transport, erythritol is introduced, a zincophilic additive, into the ZnSO_4_ electrolyte. This innovation stabilizes the Zn anode via chelation interactions between polysaccharides and Zn^2+^. Experimental tests in conjunction with theoretical calculation results verified that the erythritol additive can simultaneously regulate the solvation structure of hydrated Zn^2+^ and reconstruct the hydrogen bond network within the solution environment. Additionally, erythritol molecules preferentially adsorb onto the Zn anode, forming a dynamic protective layer. These modifications significantly mitigate undesirable side reactions, thus enhancing the Zn^2+^ transport and deposition behavior. Consequently, there is a notable increase in cumulative capacity, reaching 6000 mA h cm⁻^2^ at a current density of 5 mA cm^−2^. Specifically, a high average coulombic efficiency of 99.72% and long cycling stability of >500 cycles are obtained at 2 mA cm^−2^ and 1 mA h cm^−2^. Furthermore, full batteries comprised of MnO_2_ cathode and Zn anode in an erythritol‐containing electrolyte deliver superior capacity retention. This work provides a strategy to promote the performance of Zn anodes toward practical applications.

## Graphical abstract

Inspired by the transmembrane transport of metal ions and nutrients in cells, this study introduces a bio‐inspired electrolyte optimization strategy. Erythritol, a highly zincophilic molecule extracted from lichen, is chosen as the electrolyte additive to facilitate rapid zinc ion transport while effectively suppressing side reactions and dendrite growth.

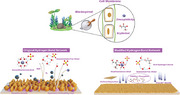

## 1 Introduction

Aqueous zinc‐ion batteries (AZIBs), consisting of a zinc anode and a Zn^2+^ redox cathode within an aqueous electrolyte, are recognized as a promising sustainable battery technology due to their inherent safety, high theoretical capacity (820 mA h g^−1^), affordability, and abundant resources.^[^ ^1^, ^2^, ^3^, ^4^ ^]^ Poised to be the next generation of large‐scale energy storage systems,^[^ ^5^, ^6^ ^]^ AZIBs, however, face significant challenges. The unstable anode‐electrolyte interface (AEI) due to side reactions and dendrite growth considerably undermines its utilization and reversibility, impacting practical applications.^[^ ^7^, ^8^ ^]^ Consequently, it is essential to find a suitable way to improve the cycling stability of Zn anodes is a critical area of focus.^[^ ^9^, ^10^, ^11^, ^12^, ^13^ ^]^

To date, substantial efforts have been made to address issues related to Zn anodes through strategies like anode structural design,^[^ ^14^, ^15^ ^]^ electrolyte formulation optimization,^[^ ^16^, ^17^, ^18^ ^]^ and the integration of artificial interfacial layers.^[^ ^19^, ^20^, ^21^ ^]^ Notably, optimizing electrolyte formulation is considered to be an effective means to improve the stability of Zn anode generally achieved through 4 types of approaches, including aqueous‐organic hybrid electrolytes,^[^ ^22^, ^23^ ^]^ highly concentrated electrolytes,^[^ ^24^, ^25^ ^]^ deep eutectic electrolytes^[^ ^26^, ^27^ ^]^ and electrolyte additives.^[^ ^16^, ^28^, ^29^, ^30^ ^]^ Among these, engineering electrolyte additives is considered a straightforward and effective method. These additives can adjust the solvation shell of hydrated Zn^2+^, weaken interactions between Zn^2+^ and free water, reduce active reaction sites, and inhibit side reactions. Additionally, certain additives are evenly adsorbed on the Zn anode to regulate the chemical environment at the AEI, preventing direct contact between free water molecules and AEI, thus inducing uniform deposition. Note that, the role of additives at the AEI in de‐solvation and nucleation is crucial for the kinetics of electrochemical reactions. For instance, Gou et al. introduced glycine to the aqueous electrolyte, resulting in its preferential adsorption onto the Zn anode and uniform deposition.^[^ ^31^ ^]^ Su et al. developed a comprehensive electrolyte additive strategy using dextrin to achieve full coverage (101) textures.^[^ ^32^ ^]^ Despite these advancements, many studies focus solely on inhibiting Zn dendrites by reshaping the solvation shell of hydrated Zn^2+^ and neglect the critical role of the AEI, leading to suboptimal deposition kinetics and reduced calendar life.^[^ ^33^, ^34^, ^35^, ^36^, ^37^ ^]^ However, these additives face challenges including mitigating single modification effects, dendrite growth, and side reactions. Hence, the development of multifunctional additives and the exploration of innovative mechanisms hold promising prospects in optimizing the electrolyte environment and modulating the AEI and Zn^2+^ transport to tackle these challenges.^[^ ^38^, ^39^ ^]^

For electrolyte optimization strategies, nature offers abundant inspiration, particularly from the transmembrane transport of ions and nutrient transportation processes in plant cells.^[^ ^28^ ^]^ Transmembrane transport is a crucial process for exchanging substances between intracellular and extracellular environments.^[^ ^40^ ^]^ It is facilitated by various channels and carrier proteins on the cell membrane, which efficiently regulate the transmembrane transport of ions, small molecules, and large molecules to maintain dynamic equilibrium within cellular internal and external environments. Polysaccharide is a kind of biomacromolecule widely present in the extracellular matrix.^[^ ^41^ ^]^ They interact with receptors and ligands on the cell membrane, participating in regulating biological processes such as cell adhesion, migration, proliferation, and differentiation. Polysaccharides enriched with polyhydroxy groups exhibiting zincophilic properties demonstrate pronounced affinity toward metal cations (Zn^2+^, Mg^2+^, Ca^2+^, Al^3+^), thereby enhancing cellular environments and improving nutrient transport efficiency.^[^ ^42^ ^]^ This phenomenon signifies that polysaccharides possess remarkable ion binding capabilities effectively regulating Zn^2+^ transportation in AZIBs to enhance their electrochemical performance and cycling stability.^[^ ^16^, ^43^, ^44^ ^]^

**Figure 1:**
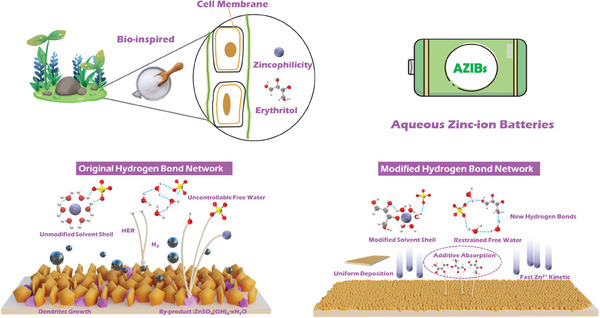
The Ert additive and the related working mechanisms on Zn anode.

Herein, inspired by the strong affinity between polyhydroxy groups and Zn^2+^, erythritol (Ert), a bio‐inspired molecule derived from lichen, has been incorporated as a functional electrolyte additive into the ZnSO_4_ electrolyte. Both experimental and theoretical calculations demonstrate that the Ert additives have admirable zincophilicity, and modulate the solvation structure through the oriented reconstruction of the hydrogen bond network. Besides, the Ert additives also preferentially absorb the Zn anode to build a dynamic interface to stabilize the Zn anode (**Figure** **1**). Based on the above principles, the Ert‐containing electrolyte extends the cycling lifespan of Zn||Zn symmetric cell (1800 h) at 1 mA cm^−2^ and 1 mA h cm^−2^. Furthermore, the Zn‐MnO_2_ full battery with Ert‐containing electrolyte also exhibits a significant capacity of 136.7 mA h g^−1^ over 1000 cycles at 1.0 A g^−1^, highlighting its promising application prospect. This study confirms that integrating Ert into the electrolyte is an effective strategy to stabilize the Zn anode, offering significant advantages for the practical development of AZIBs.

## 2 Results and Discussion

### 2.1 Investigation of the Solvation Structure in Different Electrolytes

**Figure 2:**
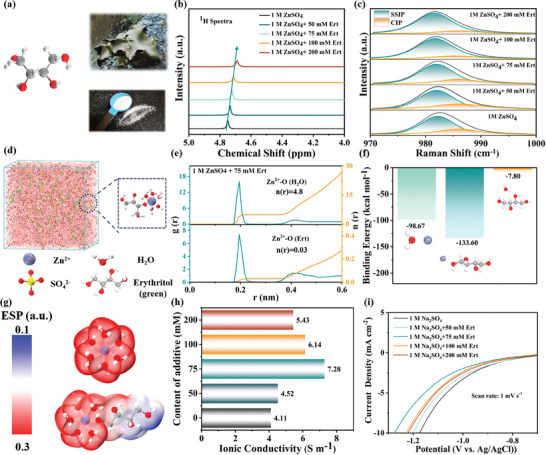
a) Structural diagram of Ert molecule extracted from lichen. b) ^1^H NMR spectra of the ZSO and Ert‐containing electrolytes. c) Raman spectra of the ZSO and Ert‐containing electrolytes. d) 3D snapshot for the Ert‐containing electrolyte system obtained from MD simulation (Ert molecules in simulation box are labeled by green color for clarity) and a partially enlarged of the [Ert‐Zn(H_2_O)_5_]^2+^ solvation structure. e) Radial distribution functions (RDFs) (g(r)) and coordination number (n(r)) of Zn^2+^‐O (H_2_O) and Zn^2+^‐O (Ert) pairs in the Ert‐containing electrolyte. f) Binding energy between different species (Zn^2+^, Ert and H_2_O). g) Electrostatic potential (ESP) distributed on the electron density van der Waals (vdW) surfaces of Zn(H_2_O)_6_^2+^ and Ert‐Zn(H_2_O)_5_^2+^. h) Ionic conductivity of the ZSO and Ert‐containing electrolytes. i) LSV curve of the ZSO and Ert‐containing electrolytes.

The Ert molecule, containing four hydroxyl groups, is extracted from lichen (**Figure** **2**a). The evolution of the solvation structure is analyzed using spectroscopy and theoretical calculations, with 1 M ZnSO_4_ as the benchmark electrolyte and Ert serving as the electrolyte additive (Figure S1, Supporting Information), respectively. Figure S2 (Supporting Information) displays the ultraviolet‐visible spectra for ZnSO_4_ electrolytes with 50, 75, 100 and 200 mm Ert (labeled as ZSO, Ert‐50, 75, 100, 200, respectively), which show a significant increase in hybrid entropy, suggesting impaired electrostatic interactions between Zn^2+^ and SO_4_.^2‐[^ ^45^ ^]^ Furthermore, as shown in Figure S3 (Supporting Information), the integration of Ert raised the pH values of the electrolytes, suggesting its role as an effective corrosion inhibitor for the Zn anode by reducing free H^+^. The local environments of the electrolytes are investigated by Nuclear Magnetic Resonance (NMR) in Figure 2b, where the ^1^H peak shifted from 4.68 to 4.75 ppm upon the addition of Ert to the ZSO electrolyte, which is ascribed to the Zn(D_2_O)_6_ formation and reduced electron density around the proton in water molecules.^[^ ^43^, ^46^ ^]^ The introduction of Ert leads to a ^1^H peak shift toward the high magnetic field, indicating a new hydrogen bond (H‐bond) network formation between Ert and H_2_O molecules. Fourier Transform Infrared (FT‐IR) spectroscopy shows a noticeable shift in the O‐H bond stretching vibration (3000–3500 cm^−1^), which demonstrates the reconstruction of the H‐bond network as depicted in Figure S4 (Supporting Information).^[^ ^27^ ^]^ Raman spectroscopy also reveals changes in the H‐bond network upon the addition of Ert molecules, with a broad *ν*(O‐H) band (2900–3800 cm^−1^) showing decreased intensity, indicating suppressed water activity.^[^ ^27^ ^]^ This is further supported by the altered proportions of water with different hydrogen bond strengths (Figures S5–S8, Supporting Information) and a distinct *ν*(C‐H) peak at 2950 cm^−1^ in the Ert‐containing electrolytes, confirming turbance of solvent molecules on H‐bond within H_2_O is stronger under similar small addition.^[^ ^43^ ^]^As shown in Figure 2c, variations in Raman peaks ranging from 960–1000 cm^−1^ are associated with *ν*(SO_4_^2−^). The classical Eigen‐Tamm mechanism suggests the association between anions and cations can be classified into two categories: solvent‐separated ion pairs (SSIP, [Zn^2+^(H_2_O)_6_‐SO_4_^2−^]) and contact ion pairs (CIP, [Zn^2+^(H_2_O)_5_‐OSO_3_^2−^]).^[^ ^47^ ^]^ A progressive decrease in the percentage of CIP occurs with increasing Ert concentration, indicating the substitution of inner‐sphere complexes in the solvation structure is regulated.^[^ ^48^ ^]^ Consequently, this adjustment reduces the interionic coupling strength between cations and anions, thereby inhibiting the formation of by‐products. These findings indicate that Ert molecules effectively modulate the solvation structure and the local chemical environment within the aqueous electrolytes.

The role of Ert is studied by molecular dynamics (MD) simulation was performed to explore the solvation structures of hydrated Zn^2+^ ions in the different electrolytes. In the ZSO electrolyte, MD simulations statistical analysis indicated that Zn^2+^ is typically encapsulated by six water molecules through Zn^2+^‐O coordination within the first solvation shell (FSS), forming a [Zn(H_2_O)_6_]^2+^ structure (Figure S9, Supporting Information). With the Ert addition, one Ert molecule is observed disrupting the FSS of [Zn(H_2_O)_6_]^2+^, replacing one coordinating water molecule and altering the solvation structure to [Ert‐ Zn(H_2_O)_5_]^2+^ (Figure 2d). Radial distribution functions (RDFs) and coordination number (n(r)) were calculated to quantify the average distribution of oxygen atoms from water or Ert around Zn^2+^. Figure 2e illustrates sharp peaks for both Zn^2+^‐O (H_2_O) and Zn^2+^‐O (Ert) approximately 2 Å from Zn^2+^, indicating Ert molecules entry into the FSS. The calculated n(r) values were 4.8 for Zn^2+^‐ O (H_2_O) and 0.03 for Zn^2+^‐O (Ert), confirming the modulation of the solvation structure for [Zn(H_2_O)_6_]^2+^ with Ert additive. Ert perturbed the original H‐bond network, leading to a reduction in H_2_O–H_2_O interactions and a decrease in the number of hydrogen bonds in the Ert‐containing electrolyte compared to the ZSO electrolyte. (Figure S13, Supporting Information).^[^ ^43^, ^49^, ^50^ ^]^ This contributed to suppressing free water reactivity. As depicted in Figure S14 (Supporting Information), mean‐squared displacement (MSD) analysis revealed higher Zn^2+^ diffusion in the Ert‐containing electrolyte (5.94 × 10^−6^ cm^2^ s^−1^) compared to the ZSO electrolyte (5.66 × 10^−6^ cm^2^ s^−1^).^[^ ^16^, ^18^, ^43^ ^]^ Density functional theory (DFT) calculations were further conducted to analyze the interaction behavior and binding energy between Zn^2+^ ion, water, and Ert molecules. As depicted in Figure 2f, the binding energy of the Zn^2+^‐Ert complex is significantly larger than that of the Zn^2+^‐H_2_O and H_2_O‐Ert complexes, proving the preference for the participation of Ert in the FSS. Electrostatic potential (ESP) distribution on the electron density van der Waals surfaces of [Zn(H_2_O)_6_]^2+^ and [Ert‐Zn(H_2_O)_5_]^2+^ revealed decreased ESP upon Ert introduction (Figure 2g), indicating reduced electrostatic repulsion around Zn^2+^ and facilitating transport. Based on these theoretical results, we can conclude that due to the strong binding affinity of Ert molecules toward Zn^2+^, Ert can effectively modulate the solvation structure of Zn^2+^ and disrupt the original H‐bond network. Consequently, water activity is diminished and the diffusion of Zn^2+^ is enhanced, promoting uniform Zn deposition and mitigating detrimental reactions induced by water decomposition. Electrochemical impedance spectra (EIS) of stainless steel symmetric cells with different electrolytes are presented in Figure 2h. Ionic conductivity in the Ert‐containing electrolytes slightly increased from 4.11 S m^−1^ to 7.28 S m^−1^ in the Ert‐75 electrolyte, but then decreased to 5.43 S m^−1^ in the Ert‐200 electrolyte. This variation confirms Ert molecule's role in enhancing Zn^2+^ ionic conductivity.^[^ ^37^ ^]^ As illustrated in Figure 2i, the electrochemical stability of these electrolytes was evaluated using linear sweep voltammetry (LSV) at the Ti working electrode. A verified suppression of the hydrogen evolution reactions (HER) due to the reduced activity of water is observed. Considering the combined effects of HER suppression and improved ionic conductivity, an optimal Ert concentration of 75 mM is obtained. Furthermore, a significant increase in the Zn^2+^ transfer number is noted, especially for the Ert‐75 electrolyte which exhibits a higher value (0.89) compared to the ZSO electrolyte (0.56), attributed to reduced electrostatic interference from SO_4_^2−^ (Figures S15–S17, Supporting Information).^[^ ^43^, ^51^ ^]^

### 2.2 Study of the Interfacial Chemistry on the Anode‐Electrolyte Interface

**Figure 3:**
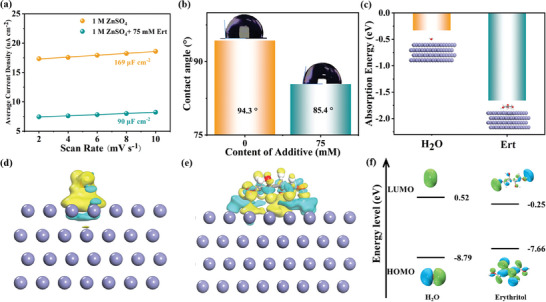
a) Average differential capacitance for Zn anode in the different electrolytes. b) The contact angle between the ZSO and Ert‐75 electrolyte on the Zn anode. c) Absorption energy comparison of H_2_O and Ert molecules on Zn (002) crystal plane, insets show the corresponding absorbed models. The 3D iso‐surfaces of charge density difference of d) H_2_O and e) Ert on the Zn (002) crystal plane (yellow and cyanine colors represent the increase and decrease of electron density, respectively). f) HOMO and LUMO energy levels of H_2_O and Ert.

It is widely assumed that additive adsorption on the Zn anode plays a significant role in inhibiting corrosion. As shown in **Figure** **3**a and Figures S18 and S19 (Supporting Information), an electric double‐layer capacitance (EDLC) analysis was conducted. The capacitance of the Ert‐75 electrolyte was measured at 90 µF cm^−2^, significantly lower than the ZSO electrolyte (169 µF cm^−2^). This marked reduction indicates the successful adsorption of Ert molecules on the Zn anode, thereby regulating Zn^2+^ electroplating behavior.^[^ ^35^ ^]^ Wettability characteristics of the ZSO and Ert‐75 electrolytes were also investigated by contact angle tests. Figure 3b shows that the contact angle decreases from 94.3° to 85.4° with the addition of Ert, indicating an increased affinity between the electrolyte and Zn anode, which facilitates reversible Zn plating/stripping at the AEI.^[^ ^37^ ^]^ FT‐IR spectra of Zn anodes immersed in the electrolyte revealed characteristic Ert‐associated peaks. As illustrated in Figure S20 (Supporting Information), broad peaks at 3300 and 1632 cm^−1^ correspond to O‐H stretching and bending vibrations, respectively. The peak at 2937 cm^−1^ is attributed to C‐H stretching vibrations, and the peak at 1049 cm^−1^ to C‐O‐C stretching vibrations. These spectral features indicate the rapid adsorption of Ert molecules, forming a protective layer on the Zn anodes to minimize direct contact with free water molecules, thus reducing dendrite growth and side reactions.

DFT calculations were employed to assess the adsorption capacities of water and Ert molecules on the Zn anode, specifically focusing on the (002) plane as the representative crystal plane. Figure 3c shows the calculated adsorption energy (E_ads_) values for Zn‐Ert and Zn‐H_2_O interactions as −1.66 and −0.33 eV, respectively. These results demonstrate a stronger affinity of Ert toward the Zn anode, which effectively impedes reactive sites for side reactions and facilitates uniform Zn deposition. Additionally, charge density difference analysis was performed to explore the interaction between the Zn anode and Ert/water molecules (Figure 3d,e). The iso‐surfaces of charge density differences suggest that electrons tend to transfer from Ert to the Zn surface during the adsorption process. Furthermore, Figure 3f demonstrates that the highest occupied molecular orbital (HOMO) energy level of the Ert molecule (−7.66 eV) is higher than the water molecule (−8.79 eV), implying a higher propensity for electron donation from Ert to the Zn anode compared to water.

### 2.3 Study on the Stability and Reversibility of Zn Anode in Different Electrolytes

**Figure 4:**
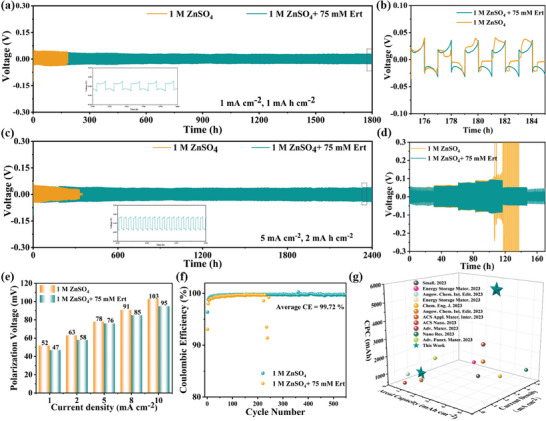
Electrochemical performances in the ZSO and Ert‐75 electrolytes. a,b) Galvanostatic charge/discharge (GCD) cycling of Zn||Zn symmetric cell at 1.0 mA cm^−2^, 1.0 mA h cm^−2^. c) GCD cycling of Zn||Zn symmetric cell at 5.0 mA cm^−2^, 2.0 mA h cm^−2^. d) Rate performance and e) the corresponding voltage hysteresis at various densities of Zn||Zn symmetric cell. f) CE test of Zn||Cu half cells. g) The comparison of CPC at various current densities with recently published results.

Given the noted advantages, the Ert‐75 electrolyte is expected to demonstrate excellent electrochemical stability and reversibility. The performance of Zn||Zn symmetric cells and Zn||Cu cells using various electrolytes were evaluated for Zn^2+^ plating/stripping capabilities. As shown in **Figure** **4**a,b, the Zn||Zn symmetric cell with the Ert‐75 electrolyte achieves a cycle life exceeding 1800 h at 1 mA cm^−2^ and 1 mA h cm^−2^, indicative of superior cycling performance. In contrast, the Zn||Zn symmetric cell with the ZSO electrolyte displays poor cycle stability and a significantly shorter life span of only 176 h. Additionally, varying Ert concentrations (50, 100, and 200 mm) resulted in a gradual decrease in cycle life. The concentration impact on performance is closely related to the formation of a compact protective layer at the AEI. At a higher current density of 5 mA cm^−2^, the Zn||Zn symmetric cell with the Ert‐75 electrolyte maintains operation for over 2400 h (Figure 4c), markedly surpassing the cell with the ZSO electrolyte. Rate performance and hysteresis voltage are presented in Figure 4d,e, with the cell in the Ert‐75 electrolyte showing lower hysteresis voltage due to the modified solvation structure and absorption layer, which accelerate Zn^2+^ kinetics.^[^ ^18^, ^43^, ^46^, ^51^, ^52^ ^]^ Notably, under harsher conditions (10 mA cm^−2^ and 4 mA h cm^−2^, Figure S21, Supporting Information), the Zn||Zn symmetric cell with the Ert‐75 electrolyte sustains over 1100 h with a cumulative plating capacity (CPC) exceeding 5500 mA h, significantly outperforming the ZSO electrolyte (230 h and 1150 mA h).

Coulombic efficiency (CE) is a critical metric for evaluating the commercial viability of the electrolyte. As exhibited in Figure 4f and Figure S22 (Supporting Information), the Zn||Cu half‐cell utilizing the Ert‐75 electrolyte exhibits a high average CE of 99.72% over 500 cycles, markedly outperforming the cell with the ZSO electrolyte, which sustains only 237 cycles. As indicated in Figure S23 (Supporting Information), the Zn||Cu cell with the Ert‐75 electrolyte attains a high initial CE of 96.39%, significantly surpassing the ZSO electrolyte. The superior electrochemical performance of Zn||Zn symmetric cells using the Ert‐75 electrolyte, excelling in comparison to several recent studies, suggests its potential application in AZIBs (Figure 4g).^[^ ^16^, ^17^, ^27^, ^29^, ^31^, ^37^, ^43^, ^53^, ^54^, ^55^, ^56^ ^]^

### 2.4 The Morphology Evolution of Zn Anodes in Different Electrolytes

**Figure 5:**
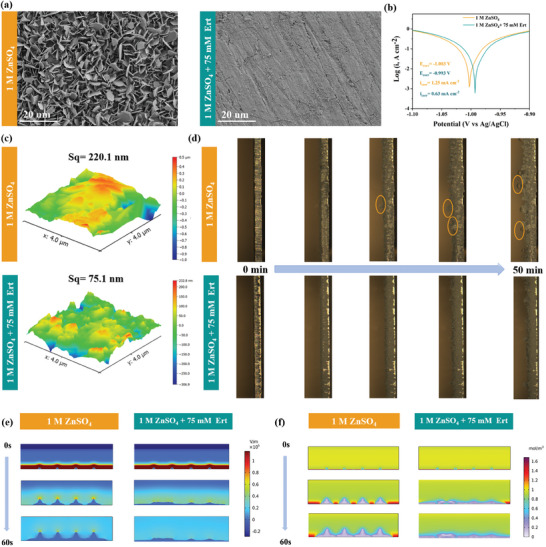
Surface morphology evolution of Zn anode in the ZSO and Ert‐75 electrolytes. a) SEM images and b) Tafel plots of the Zn foils in the ZSO and Ert‐75 electrolytes at 10 mV s^−1^ based on a three‐electrode system. c) AFM images of Zn anode after 50 cycles at 2 mA cm^−2^ and 1 mA h cm^−2^. d) In situ optical observations of Zn deposition morphologies. COMSOL Multiphysics simulation of Zn anode during plating for **e**) electric field and f) Zn^2+^ flux.

To assess the effectiveness of the Ert additive in promoting efficient and uniform Zn plating while reducing side reactions and the formation of “dead zinc”, a morphological analysis of the Zn anode was performed. As shown in **Figure** **5**a, a rough Zn deposition characterized by dendrites is evident after 50 cycles in the ZSO electrolyte. In sharp contrast, a uniform and shiny Zn deposition can be obtained in the Ert‐75 electrolyte. This result is also supported by X‐ray diffraction (XRD) patterns (Figure S27, Supporting Information), where several undesired diffraction peaks corresponding to the Zn_4_SO_4(_OH)_6_·xH_2_O by‐products are detected in the ZSO electrolyte, whereas no obvious diffraction peaks corresponding to such by‐products are observed in the Ert‐75electrolyte. This suggests that Ert molecule adsorption on the Zn anode influences the preferred orientation for uniform horizontal growth.^[^ ^57^ ^]^ As depicted in Figure S24 (Supporting Information), the cell in the ZSO electrolyte exhibited rough Zn deposition with random dendrites after deposition. In sharp contrast, a uniform and flake‐like pattern emerged in the Ert‐75 electrolyte, also supported by XRD patterns (Figure S25, Supporting Information). Tafel plots displayed in Figure 5b demonstrate the significant anti‐corrosion effect of the Ert‐75 electrolyte. The corrosion potential shifted from −1.003 V in the Ert‐75 electrolyte to −0.993 V in the ZSO electrolyte, indicating that Ert molecules effectively mitigate self‐corrosion. Atomic force microscopy (AFM) images in Figure 5c reveal that the Zn anode cycled in the ZSO electrolyte has a higher root mean square (S_q_), compared to the smoother in the Ert‐75 electrolyte after cycling. These observations are further visually substantiated by confocal laser scanning microscopy images in Figure S26 (Supporting Information).

As shown in Figure 5d, in situ optical microscopy was employed to capture real‐time images of Zn anodes at a current density of 2 mA cm^−2^. In the ZSO electrolyte, disordered protrusions formed on the surface of the Zn anode within 20 min, intensifying over time due to the “tip effect”, leading to uneven Zn deposition and dendrite formation. After 50 min, a significant number of bubbles due to HER were observed at the AEI. In contrast, uniform Zn deposition was consistently observed in the Ert‐75 electrolyte throughout the plating process. XRD analysis of Zn anodes after seven days of immersion in the two electrolytes (Figure S28, Supporting Information) revealed unsatisfactory peaks associated with by‐products (Zn_4_(OH)_4_(SO_4_)_2_·xH_2_O) formation in the ZSO electrolyte, while no significant changes were observed in the Ert‐75 electrolyte. These findings underscore the crucial role of the Ert additive in promoting uniform Zn deposition, inhibiting parasitic side reactions, and enhancing anti‐corrosive properties. Finite element analysis conducted by COMSOL software shows that during the plating process, irregular micro‐bumps are typically formed on the Zn anode due to its uneven roughness. In the ZSO electrolyte, a distinct intensity gradient in the spatial distribution of electric fields was observed on the Zn anode, ranging from isolated nuclei to encompassing the entire surface, increasing with deposition time (Figure 5e). The localized electric field accelerates dendrite evolution by accumulating excessive charges at bump tips. However, Ert additives with zincophilic properties can effectively inhibit dendrite growth after adsorption at the AEI, regulate electric field intensities, and efficiently deposit on concave surfaces. Furthermore, zincophilic additives ensure effective regulation of Zn^2+^ flux distributions so that deposition rates vary according to position (Figure 5f); The Zn anode with uniform and compact Zn deposition gradually fills the entire concave surfaces.

### 2.5 Assessment of the Deposition and Diffusion Kinetics of Zn^2+^ in Different Electrolytes

**Figure 6:**
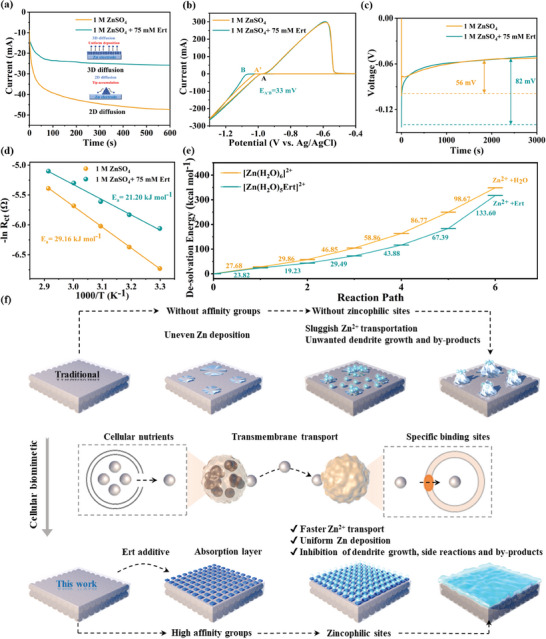
a) CA curves of Zn anode at 150 mV in the ZSO and Ert‐75 electrolytes. b) CV curves of Zn^2+^ nucleation on Ti foil at 10 mV s^−1^. c) Nucleation overpotential of Zn anode in the ZSO and Ert‐75 electrolytes based on the Zn||Zn symmetric cells. d) The *E_a_* values were obtained from Nyquist plots using Zn||Zn symmetric cells in the ZSO and Ert‐75 electrolytes. e) The calculated de‐solvation energies for the stepwise [Zn(H_2_O)_6_]^2+^ and [Ert‐Zn(H_2_O)_5_]^2+^ de‐solvation process. f) Schematic diagram of Zn^2+^ nucleation and growth mechanism in the ZnSO_4_ electrolyte without/with Ert additive.

To further explore the deposition and diffusion kinetics in different electrolytes, chronoamperometry (CA) measurements were conducted at an applied overpotential of −150 mV. **Figure** **6**a reveals that in the ZSO electrolyte, a continuous increase in current over 300 s was observed, indicative of uncontrollable 2D diffusion of Zn^2+^ associated with the “tip effect”. In contrast, the Zn anode in the Ert‐75 electrolyte shows a moderate current plateau, suggesting a dominance of 3D diffusion during nucleation.^[^ ^19^, ^58^, ^59^, ^60^ ^]^ Additionally, in the Ert‐75 electrolyte, a reduced nucleation rate and suppressed diffusion behavior were noted, attributed to Ert molecules promoting the dehydration of [Zn(H_2_O)_6_]^2+^, thus accelerating the reaction rate and limiting 2D diffusion of Zn nuclei.^[^ ^35^, ^61^, ^62^, ^63^ ^]^ The overpotential corresponding to initial nucleation states was analyzed to quantify the Ert molecule's effect on Zn deposition. A lower overpotential was achieved in the Ert‐75 electrolyte for Zn nucleation compared to the ZSO electrolyte (Figure 6b), suggesting that the adsorption of Ert at the AEI forms an electrostatic shielding layer, creating a slower energy barrier of finer Zn nuclei. This observation aligns with the voltage curves in Zn||Zn symmetric cells at 2 mA cm^−2^ (Figure 6c), further corroborating recent findings.^[^ ^18^, ^35^, ^43^, ^55^ ^]^

To assess the impact of the Ert adsorption layer on the solvation structure and H‐bond network, activation energy (*E_a_*) was determined using electrochemical impedance response testing at various temperatures. As depicted in Figure 6d and Figure S30 (Supporting Information), by applying the Arrhenius equation and analyzing the relationship between the reciprocal of temperature and charge transfer resistance (*R_ct_*), an *E_a_* of 23.85 kJ mol^−1^ was obtained in the Ert‐75 electrolyte, which is lower than the 28.82 kJ mol^−1^ in the ZSO electrolyte. This result indicates that the Ert additive can reduce the energy barrier of [Zn(H_2_O)_6_]^2+^ cluster during the de‐solvation process.^[^ ^51^, ^52^, ^55^ ^]^ As indicated in Figure 6e, the de‐solvation energy for [Zn(H_2_O)_6_]^2+^ is higher than that of [Ert‐Zn(H_2_O)_5_]^2+^, implying that Ert additive can effectively accelerate peeling off the outer solvation sheath hydrated Zn^2+^ at the AEI.^[^ ^51^, ^55^ ^]^ Both experimental and theoretical analyses substantiate that the incorporation of Ert additive effectively mitigates energy consumption, which is in favor of Zn^2+^ transport kinetics.

In combination with experimental and theoretical calculations, the optimization mechanisms of the Ert additive are depicted in Figure 6f. In the traditional ZSO electrolyte, the deposition process inevitably involves a de‐solvation penalty step that eliminates the solvation shell of hydrated Zn^2+^, resulting in the generation of numerous free water molecules at the AEI. Furthermore, the presence of minute protrusions on Zn anodes induces uneven Zn deposition, ultimately giving rise to dendrites and byproducts. To overcome these challenges, a zincophilic additive is employed for rapid adsorption at the AEI and the formation of a protective layer. This bio‐inspired approach facilitates Zn^2+^ transport, enhances cycling performance, promotes de‐solvation processes, and inhibits parasitic reactions.

### 2.6 Evaluation of Full Batteries Electrochemical Performance in Various Electrolytes

**Figure 7:**
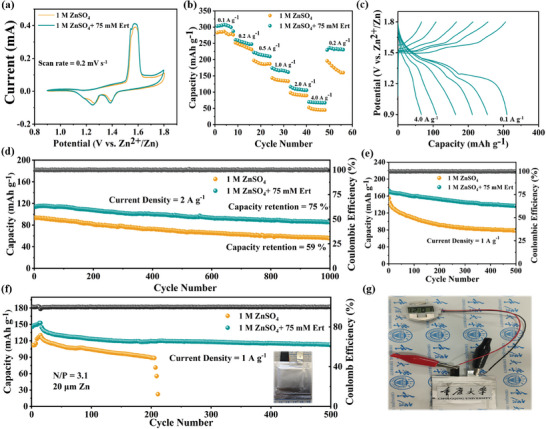
Electrochemical performance of Zn||δ‐MnO_2_ full batteries in the ZSO and Ert‐75 electrolytes. a) CV curves. b) Rate performance. c) GCD curves for Zn||δ‐MnO_2_ full battery at different current densities in the Ert‐75 electrolyte. d) Cycling performance of Zn||δ‐MnO_2_ full battery tested at the current density of 2.0 A g^−1^ and e) 1.0 A g^−1^. f) Cycling performance of Zn||δ‐MnO_2_ pouch battery tested at the current density of 1.0 A g^−1^. g) Photograph of Zn||δ‐MnO_2_ pouch battery.

To evaluate practical applications of the Ert‐containing electrolyte, δ‐MnO_2_ was coupled to construct full batteries and characterized by SEM and XRD, respectively (Figures S31 and S32, Supporting Information). As demonstrated in **Figure** **7**a, the cell assembled with the Ert‐75 electrolyte exhibits a higher current density. The rate performance of the Zn||MnO_2_ full battery was evaluated at various current densities. As depicted in Figure 7b, the full battery with the Ert‐75 electrolyte achieves a high discharge capacity of 307.1 mA h g^−1^ at 0.1 A g^−1^, with specific capacities of 254.2, 219.9, 170.7, 112.4, and 68.9 mA h g^−1^ maintained at 0.2, 0.5, 1.0, 2.0, and 4.0 A g^−1^, respectively. The GCD curves for these capacities are presented in Figure 7c and Figure S33 (Supporting Information). The Zn||MnO_2_ full battery with the Ert‐75 electrolyte retains a capacity of 85.2 mA h g^−1^ after 1000 cycles, corresponding to 75.2% capacity retention at 2.0 A g^−1^ (Figure 7d). In contrast, the battery with the ZSO electrolyte demonstrates a rapid capacity decrease, retaining only 55.1 mA h g^−1^ and 59.3% capacity after the same period. Additionally, at 1.0 A g^−1^, the Zn||MnO_2_ full battery with the Ert‐75 electrolyte also exhibits exceptional long‐term cycling stability (Figure 7e). As shown in Figure 7f, even under the practical applications with an ultrathin Zn anode (20 µm) displays superior long‐term cycling performance, showing a decent capacity of 108.2 mA h g^−1^ after 500 cycles at 1.0 A g^−1^_._ The result presented in Figure 7g demonstrates the efficient power supply capability of the pouch battery when used to operate a digital timer, highlighting the effectiveness of Ert in mitigating parasitic reactions and minimizing energy dissipation. SEM images shown in Figure S34 (Supporting Information) reveal that the Zn anode worked in the Ert‐75 electrolyte exhibits a uniform and compact deposition surface after 100 cycles, unlike the ZSO electrolyte, which displays a rougher surface characterized by disorganized dendrite accumulation. The self‐discharge test was assessed by subjecting batteries to a charging step up to 1.8 V, followed by a resting period of 24 h, and subsequently discharging down to 0.9 V. As illustrated in Figure S35 (Supporting Information), the Zn||MnO_2_ full battery with the Ert‐75 electrolyte retained 92.7% of its initial capacity, surpassing the 86.8% retention observed for batteries with the ZSO electrolyte. Notably, the *R_ct_* value for a full battery in the Ert‐75 electrolyte is much smaller, indicating that the Ert‐75 electrolyte can effectively boost the Zn^2+^ migration kinetics (Figure S36, Supporting Information). These findings highlight how employing Ert as an electrolyte can lead to enhanced battery performance and improved stability compared to conventional alternatives such as ZSO electrolyte.

## 3 Conclusion

In conclusion, Ert, a green and bio‐inspired additive enriched with polyhydroxy groups, has been demonstrated to significantly enhance the performance of AZIBs. Ert contributes to the aqueous electrolyte in several crucial ways, it effectively reduces the activity of primitive H_2_O molecules by reconstructing the H‐bond network and modulating solvent structure, while also forming a protective layer. These actions synergistically suppress dendrite growth, side reactions, and corrosion. The Zn||Zn symmetric cell using the Ert‐75 electrolyte demonstrated a prolonged lifespan of over 1800 h at 1 mA cm^−2^ and 1 mA h cm^−2^. Notably, under a higher current density of 5 mA cm^−2^, the Zn||Zn symmetric cell maintains excellent cycling stability for >2400 h. Furthermore, the constructed full battery exhibited an impressive lifespan, sustaining 1000 cycles with a capacity retention of 75.2%. This strategy of utilizing Ert as an additive holds significant potential for developing high‐performance AZIBs.

## Conflict of Interest

The authors declare no conflict of interest.

## Supporting information

Supporting Information

## Data Availability

Research data are not shared.
