# Supplementary material for: Co‐Regulating Solvation Structure and Hydrogen Bond Network via Bio‐Inspired Additive for Highly Reversible Zinc Anode
Source: Adv Sci (Weinh). 2024 Jul 21;11(35):2404968. doi: 10.1002/advs.202404968 (PMC11425239; doi:10.1002/advs.202404968)
Supplement: Supplementary file 1 — Supporting Information [file ADVS-11-2404968-s001.docx]

**Co-regulating Solvation Structure and Hydrogen Bond Network via Bio-inspired Additive for Highly Reversible Zinc Anode**

*Sida Zhang^ac#^, Qianzhi Gou^bcf#^, Weigen Chen^ac^*, Haoran Luo^bc^, Ruduan Yuan^bc^, Kaixin Wang^bc^, Kaida Hu^ac^, Ziyi Wang^ac^, Changding Wang^ac^, Ruiqi Liu^ac^, Zhixian Zhang^e^, Yu Lei**^ac^_,_ Yujie Zheng^bc^, Lei Wang^bc^, Fu Wan^ac^_,_ Baoyu Li^d^* and Meng Li^bc^**

1. State Key Laboratory of Power Transmission Equipment Technology, School of Electrical Engineering, Chongqing University, Chongqing 400044, China
2. MOE Key Laboratory of Low-grade Energy Utilization Technologies and Systems, CQU-NUS Renewable Energy Materials & Devices Joint Laboratory, School of Energy & Power Engineering, Chongqing University, Chongqing 400044, China
3. National Innovation Center for Industry-Education Integration of Energy Storage Technology, Chongqing University, Chongqing 400044, China
4. State Key Laboratory of Radiation Medicine and Protection, School for Radiological and Interdisciplinary Sciences (RAD-X) and Collaborative Innovation Center of Radiation Medicine of Jiangsu Higher Education Institutions, Soochow University, Suzhou 215123, China
5. School of Electrical and Electronic Engineering, Chongqing University of Technology, Chongqing 400054, China
6. School of Building Services Science and Engineering, Xi'an University of Architecture and Technology, Xi'an, 710055, China

^#^ These authors contributed equally to this work

^*^ *Corresponding Email: weigench@cqu.edu.cn; limeng@cqu.edu.cn; byli@suda.edu.cn*

**Materials:**

Zinc sulfate heptahydrate (ZnSO_4_·7H_2_O, alfa, AR, > 99.9%); Erythritol (C_4_H_10_O_4_, Energy Chemical, AR, 99%); Zn foil (> 99.9%, thickness: 20 and 50 μm); Cu foil (thickness: 30 μm); Ti foil (thickness: 30 μm) ; Glass fiber membrane (Whatman, model: GF/D); KMnO_4_ (0.948 g, Energy Chemical, AR, 99%); MnSO_4_ (0.169 g, Energy Chemical, AR, 99%) were obtained from Shanghai Titan Technology. All chemical reagents are in analytical grade and used as received. Besides, the zinc foil is polished by 1500 grit sandpaper before using in order to remove the passivation layer. The deionized (DI) water was obtained from an ultra-pure purification system in our lab.

**Electrolyte preparation:**

At first, 1 M ZnSO_4_ aqueous electrolyte was acquired by dissolving zinc sulfate heptahydrate in DI water. Subsequently, various amounts of erythritol (50 mM, 75 mM, 100 mM and 200 mM) additive were introduced into the 1 M ZnSO_4_ electrolyte to obtain the uniform hybrid electrolyte with 1 h magnetic stirring, respectively.

**Synthesis of** **δ-MnO_2_ cathode:**

δ-MnO_2_ was synthesized via a simple hydrothermal method. In detail, KMnO_4_ (0.948 g, Energy Chemical, AR, 99%) and MnSO_4_ (0.169 g, Energy Chemical, AR, 99%) were dissolved in 20 ml DI water. The obtained solutions were then put into a 50 mL Teflon-lined stainless autoclave and maintained at 160 °C for 12 h. Finally, the resulting black precipitates were centrifuged, washed with DI water for several times, and dried in a vacuum at 60 °C for 12 h. And the cathodes were prepared via mixing the commercial δ-MnO_2_ cathode, conductive carbon (Canrd) and PVDF (Canrd) at the mass ratio of 8:1:1, and then coating the slurry on Ti foil. The average mass loading of the active material was about 4.00 mg cm^−2^. After drying under vacuum at 60 °C for 12 h, cutting into round electrode pieces with a diameter of 12 mm.

**Cells fabrication:**

Zn||Zn symmetric cells were assembled by sandwiching the glass fiber (separators) between commercial Zn foil (diameter:12 mm). Zn||Cu cells were assembled using commercial Zn foil (diameter: 12 mm) as anode, Cu foils (diameter: 12 mm) as cathode, and glass fiber (GF/D, Whatman) as separator (diameter: 16 mm). Zn||MnO_2_ full cells were assembled using Ti foil (diameter: 16 mm), Zn foil and glass fiber as cathode collector, anode and separator, respectively. All the coin cells were assembled in CR2032 with the different type electrolyte (50 μL cm^-2^)

For the manufacture of soft pack batteries, we provide the following details:

Anode: Zinc foil (20 μm)

Anode mass: 0.187 g

Cathode: MnO_2_

Collector: Ti foil (30 μm)

Cathode area: 20 cm^2^

Areal density (active material): 4.00 mg cm^-2^

N/P ratio refers to the excess capacity of the anode over the cathode under the same conditions. *Q_NE,_ Q_PE_, q_NE_, q_PE_, m_NE_, m_NE_* represents the anode capacity. cathode capacity. anode theorical capacity, cathode theorical capacity, anode mass and cathode mass, respectively.

$$N/P=Q_{NE}/Q_{PE}=\frac{q_{NE}\cdot m_{NE}}{q_{PE}\cdot m_{PE}}=\frac{187\cdot10^{-3}\cdot820}{80\cdot10^{-3}\cdot616}=3.1$$

**Characterizations:**

The crystal structure and phase composition of the as-obtained electrodes was characterized by X-ray diffraction (XRD, Rigaku Ultima IV diffractometer with Cu K radiation). The morphology was conducted by scanning electron microscopy (SEM. Sigma 500) and 3D measuring laser microscope. The vibration of various electrolytes were collected by Raman spectroscopy (DXR, Thermo Fischer). The solvation structure of various electrolyte was verified by the Fourier Transform infrared (FT-IR) spectra (Nicolet iS5, Thermo Scientific) and nuclear magnetic resonance spectrometer (1H NMR, AVANCE III HD600MHz), pure D_2_O replaces DI water as the solvent. The pH values of various electrolytes were collected by a MP511 pH meter, Sanxin. The roughness of cycled-Zn plates with different electrolytes was recorded by using an atomic force microscope (AFM, Bruker) and laser confocal microscopy (Leica, TCS SP8). The in-situ microscope images for Zn deposition process were obtained by commercial high-resolution camera (YUESCOPE Dendrite Observation Optical System) equipped a magnifying glass holder.

**Electrochemical performance:**

Galvanostatic charge-discharge (GCD) tests were conducted using the Lanhe CT-3002A battery test system while all electrochemical tests were carried out with the bio-logic electrochemical workstation. Cycling performance was carried out 1, 5 and 10 mA cm^-2^, and the corresponding areal capacity is 1, 2, and 4 mA h cm^-2,^ respectively. The rate performance of Zn||Zn symmetric cells with different electrolyte was measured at 1, 2, 5, 8, and 10 mA cm^-2^ with an areal capacity of 1.0 mA h cm^-2^. The Coulombic efficiency (CE) measurements were conducted on asymmetrical Zn||Cu cells under 2 mA cm^-2^, 1 mA h cm^-2^ and the cutoff potential is 0.5 V. Abbreviations for technical terms were explained upon their first use. Utilizing the three-electrode system, linear sweep voltammetry (LSV) was employed at a rate of 2 mV s^-1^ to determine the electrochemical potential windows for different electrolytes, with Ti foil, Pt plate, and Ag/AgCl utilized as the working electrode (WE), counter electrode (CE), and reference electrode (RE), respectively. To avoid the influence of Zn^2+^ deposition behaviour, we selected Na_2_SO_4_ and Na_2_SO_4_+Ert solutions as testing solutions.

We tested the ionic conductivities of various electrolytes using stainless steel (SS) symmetric cells, and calculated them using the equation: 𝜎 = 𝑙/(𝑅_S_·𝑆), where 𝑙 represents the distance between two SS electrodes, 𝑅_S_ represents the resistance from an electrochemical impedance spectrum test, and 𝑆 is the area of blocking electrodes. When first introduced, technical term abbreviations were explained. The contact area with the electrolyte is 1.13 cm^-2^, and the two SS electrodes are separated by around 200 μm. Equation C=-(ωZ_im_)^-1^ was used to acquire the differential capacitance curves. Here, C, ω, and Z_im_ represent the differential capacitance, angular frequency, and imaginary part of impedance, respectively.

To evaluate the anti-corrosion properties, the Tafel plot was measured at a rate of 1 mV s^-1^ using a three-electrode system configuration. The working electrode comprised of Zn foil, while the counter electrode was made of Ti foil, and Ag/AgCl was utilized as the reference electrode. The Pt plate functioned as the counter electrode, and the Ag/AgCl electrode was employed as the reference. The Pt plate functioned as the counter electrode, and the Ag/AgCl electrode was employed as the reference. In addition, the chronoamperometry (CA) measurements were carried out using a Zn||Zn symmetric cells. Finally, a standard three-electrode set-up consisting of Ti foil (WE), Pt plate (CE), and Ag/AgCl (RE) is employed to compare the nucleation overpotential of Zn^2+^ in different electrolytes.

To examine Zn stripping and plating behavior, Zn||Zn symmetric cells were built within CR2032 coin cells containing commercial Zn foil and a 1 M ZnSO_4_ electrolyte, with or without the Ert additive. It is important to note that the charge cutoff voltage for all symmetric cells is set at 0.5 V. The cycling performance was evaluated at 1, 5 and 10 mA cm^-2,^ with corresponding areal capacity of 1, 2 and 4 mA h cm^-2^, respectively. The performance rate of Zn||Zn cells using various electrolytes was quantified at 1, 2, 5, 8 and 10 mA cm^-2^ with an areal capacity of 0.5 mA h cm^-2^. The Coulombic efficiency (CE) was measured using asymmetric Zn||Cu half cells under 2 mA cm^-2^, 1 mA h cm^-2^ with a cutoff potential of 0.5 V. The full cell electrochemical performance was assessed using the Zn-MnO_2_ battery, incorporating CR2032 cells and pouch cells (3 cm × 4 cm). Zn^2+^ transference number (t_Zn_) can be deduced form Bruce–Vincent–Evans equation as follows

$$t_{+}=\frac{I_{ss}\left(\Delta V-I_{0}R_{0}\right)}{I_{0}\left(\Delta V-I_{ss}R_{ss}\right)}$$

Where ∆V is the applied polarization voltage (20 mV), *I_0_* and *R_0_* are the initial current and interfacial resistance before polarization, respectively. *I_SS_* and *R_SS_* are the steady-state current and interfacial resistance after polarization for 3600s, respectively

**Computational methods**

**All-atom molecular dynamics (MD) simulations**

In all simulations, 75 Ert molecules were randomly dispersed among 1000 ZnSO_4_ molecules. The Ert-ZnSO_4_ complex was then solvated in a 10 × 10 × 10 nm^3^ water box (containing 27427 water molecules) with periodic boundary conditions applied to all three directions. Water molecules were represented using the SPC/E model. All MD simulations were carried out with the GROMACS software package using the AMBER99SB force field. The long-range electrostatic interactions were handled using the particle mesh Ewald (PME) method (using a fourth-order interpolation and a maximum Fourier spacing of 0.1 nm), whereas the van der Waals (vdW) interactions were handled with a cutoff distance of 1.0 nm. The bond length involving hydrogen atoms was constrained with the LINCS algorithm. The system was first energy minimized with restraints imposed on the positions of the heavy atoms. Subsequently, the system was pre-equilibrated for 0.5 ns in the NVT ensemble using a ν-rescale thermostat at 300 K. Following this, 10 ns production simulations were carried out at a constant pressure of 1 bar and a temperature of 300 K using the Berendsen coupling method. Moreover, a control system consisting of 1000 ZnSO_4_ molecules but lacking Ert was modeled for comparison ^[1]^.

**Quantum chemistry calculations**

Density functional theory (DFT) calculations were performed using the Gaussian 09 program to obtain the binding energies of Zn^2+^-H_2_O, Zn^2+^-Ert and H_2_O-Ert, as well as the de-solvation energies of [Zn(H_2_O)_n_]^2+^ (n = 1, 2, 3, 4, 5 and 6) and [Ert-Zn(H_2_O)_n_]^2+^ (n = 1, 2, 3, 4 and 5). Additionally, the electrostatic potential (ESP) of [Zn(H_2_O)_6_]^2+^ and [Ert-Zn(H_2_O)_5_]^2+^, and the iso-surface maps for the highest occupied molecular orbital (HOMO) and the lowest unoccupied molecular orbital (LUMO) of H_2_O and Ert molecules were also calculated. The geometries were optimized at the B3LYP-D3/6-311+G** level. For each optimized compound, single-point energies were calculated at the B2PLYP-D3/Def2TZVPP level to obtain the binding energies (E_b_) of Zn^2+^-H_2_O, Zn^2+^-Ert and H_2_O-Ert complexes. The de-solvation energies for [Zn(H_2_O)_n_]^2+^ (n = 1, 2, 3, 4, 5 and 6) were calculated according to the following equation^[2,3]^:

$$E_{desol-n}=\cdot E([Zn(H_{2}O)_{n-1}]^{2\text{+}})\cdot+\cdot E(H_{2}O)\cdot-\cdot E([Zn(H_{2}O)_{n}]^{2\text{+}})$$

The de-solvation energies for Ert-Zn(H_2_O)_n_^2+^ (n = 1, 2, 3, 4 and 5) were calculated according to the following equations:

$$E_{desol-Ert-n}=\cdot E([Ert-Zn(H_{2}O)_{n-1}]^{2+})\cdot+\cdot E(H_{2}O)\cdot-\cdot E([Ert-(H_{2}O)_{n}]^{2+})$$

The ESP of [Zn(H_2_O)_6_]^2+^and [Ert-Zn(H_2_O)_5_]^2+^, as well as the iso-surface maps of the HOMO and LUMO of H_2_O and Ert molecules were plotted by VMD software based on Muitiwfn exported cube files.

First-principles calculations were carried out to obtain the adsorption energies of H_2_O and Ert molecules and the charge density difference of H_2_O and Ert molecules along the (002) plane of Zn anodes with the corresponding iso-surface using DMol^3^. A 6 × 6 × 4 Zn slab with a vacuum spacing of 20 Å was used to represent the absorbed surface for H_2_O and Ert molecules. The generalized gradient approximation (GGA) with the Perdew-Burke-Ernzerhof (PBE) function was used as the exchange-correlation function. For treating the long-range van der Waals interactions, we employed the empirical correction in the Grimme scheme. The DFT Semi-core Pseudopots (DSPP) and double numerical plus polarization (DNP) basis set were adopted for the Zn and C/H/O atoms, respectively. The Zn atoms in the bottom two layers of Zn slab were kept fixed during geometrical optimizations, and the total energy change and displacement were set to 2.0 × 10^-5^ Ha and 2.0 × 10^-3^ Å respectively. The adsorption energies of H_2_O and Ert molecules along Zn anodes were calculated as follow:

$$E_{ads}=E_{(total)}-E_{(surface)}-E_{(molecule)}$$

where *E_(total)_* is the total energy of H_2_O/Ert adsorbed on slab, *E_(surface)_* and *E_(molecule)_* are the total energies of Zn slab and single isolated H_2_O/Ert molecules, respectively.

**Finite Element Analysis (FEA).**

In the anode unit of a Zn||Zn symmetric cell, the phase field variables satisfy the Butler-Volmer equation and also comply with the following equations^[4]^:

Where *ξ* (electrolyte, *ξ*= 0; zinc metal anode, *ξ = 1*); L_σ_ is the interface mobility; L_η_ is the reaction constant; α and 1−α are the charge transfer coefficients; c0 is the initial concentration of the electrolyte; *H(ξ) = ξ^3^(6ξ^2^−15ξ+10)* is the interpolation function; *η = φ_Zn_−φ_e_−E_eq_* is the overpotential, where *φ_Zn_* is the potential of the zinc metal anode, *φ_e_* is the potential of the electrolyte, and *E_eq_* is the potential at the electrochemical reaction equilibrium.

The evolution of *c_Zn2+_* in the electrolyte can be described using the Nernst-Planck equation:

Where *D_Zn_^2+^* is the diffusion coefficient of Zn^2+^, and c_Zn_ is the initial concentration at the electrode.

Geometry structure:

The model proposes a coupled mechanical-electrochemical phase field approach to replicate the multi-physics processes in the Zn||Zn symmetric cell. The two-dimensional domain used consists of an electrode with Zn metal nucleation sites and an electrolyte. The initial geometry structure is depicted in the following figure, where the electrolyte is in liquid form.

Model assumptions:

Neglect the opposing cathode where zinc stripping reactions occur; Assume the electrolyte is a concentration constant bulk solution; Due to the electrolyte being either flowable or deformable, assume the Zn-electrolyte interface is in perfect contact after battery encapsulation.

Boundary conditions:

In the anode unit of the Zn||Zn symmetric cell, a constant voltage of 50 mV is applied as the driving force; The boundary load on the upper boundary of the anodic unit in the Zn||Zn symmetric cell is set to 5×10^-6^ Pa; The boundary load on the upper boundary of the additive is set to 4×10^-7^ Pa.

Model parameters:

The parameters required for the calculation of the anode unit model in a Zn||Zn symmetric cell are shown in **Tab. S1**

**Tab. S1 Model parameters required for the calculation**

| Structural parameter | Height (μm) | 3 |
| --- | --- | --- |
|  | Width (μm) | 11.25 |
| Physical parameter | Ambient temperature (K) | 298.15 |
|  | Electrolyte diffusion coefficient (m^2^/s) | 2×10^-15^ |
|  | Electrode diffusion coefficient (m^2^/s) | 2×10^-15^ |
| Material parameters | Conductivity of zinc metal (S/m) | 1.6×10^-8^ |
|  | Zinc metal measuring point density (mol/m^3^) | 109667 |
|  | Electrolyte conductivity (S/m) | 0.1 |
| Operation parameters | Electrochemical reaction rate constant (1/s) | 0.5 |
|  | Interface migration rate (m^3^/(J·s)) | 10^-6^ |
|  | Initial electrolyte concentration (mol/L) | 1 |

**Fig. Section**


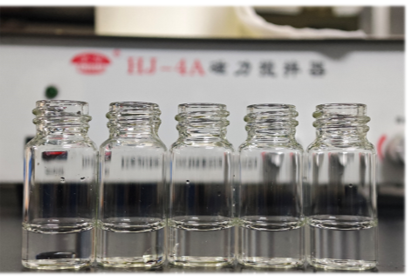


**Fig. S1** Optical images of the ZSO electrolytes with various concentrations Ert additive (from left to right：0, 50, 75, 100 and 200 mM).





**Fig. S2** UV-vis spectrum of ZnSO_4_ electrolytes with various concentrations of Ert additives.





**Fig. S3** The pH values of ZnSO_4_-based electrolytes with various content of Ert additives.

**.**
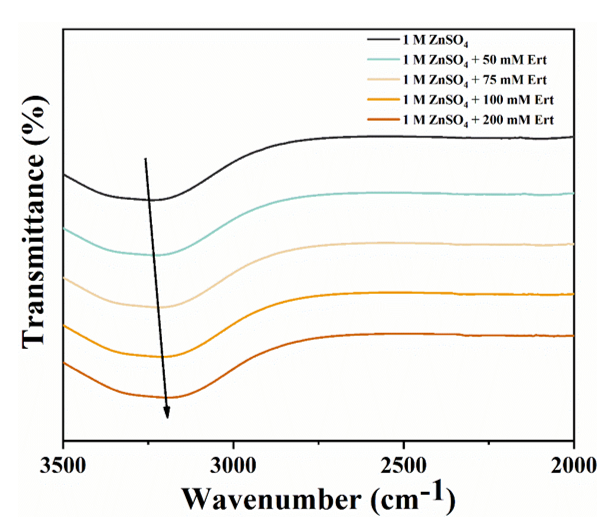


**Fig. S4** FT-IR spectra of electrolyte system with various content of Ert additives.


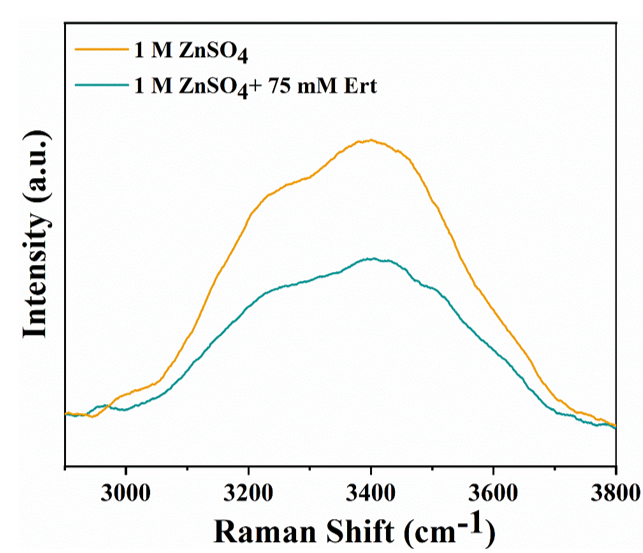


**Fig. S5** Raman spectra of the ZSO and Ert-75 electrolytes.

**

**

**Fig. S6** The Raman of the ZSO electrolyte was fitted with three peaks representing the O–H stretching vibration of H_2_O in weak, medium, strong H-bond state.





**Fig. S7** The Raman of the Ert-75 electrolyte was fitted with three peaks representing the O-H stretching vibration of H_2_O in weak, medium, strong H-bond state.


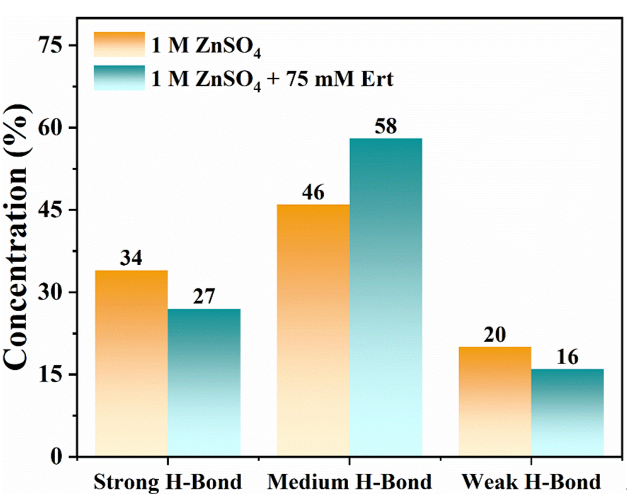


**Fig. S8** Summary proportion of H-bond with different states in the ZSO and Ert-75 electrolytes.

**
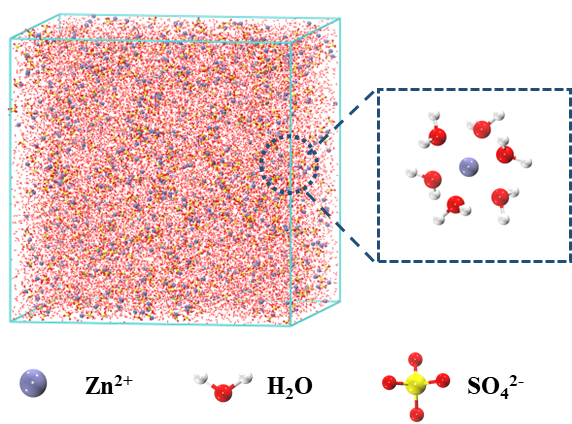
**

**Fig. S9** Three-dimensional snapshot for the pristine ZnSO_4_ electrolyte system obtained from MD simulation and a partially enlarged of the [Zn(H_2_O)_6_^2+^] solvation structure.





**Fig. S10** g(r) and n(r) of Zn^2+^-O (H_2_O) in the pristine ZnSO_4_ electrolyte.


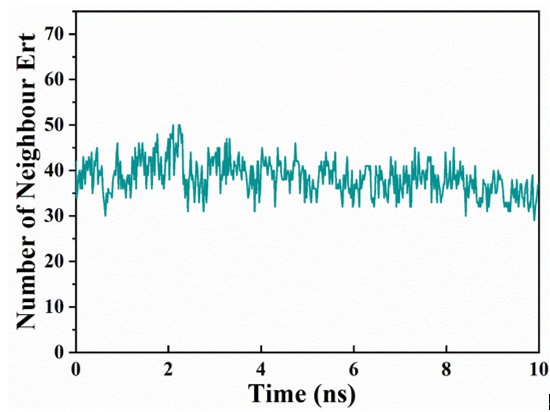


**Fig. S11** Time-resolved number of Ert within the distance of 4 Å away from central Zn^2+^, the total number of Ert molecules in the system is 75.





**Fig. S12** Coordination number (n(r)) of Zn^2+^-O (H_2_O) pairs in the ZSO and Ert-75 electrolytes.


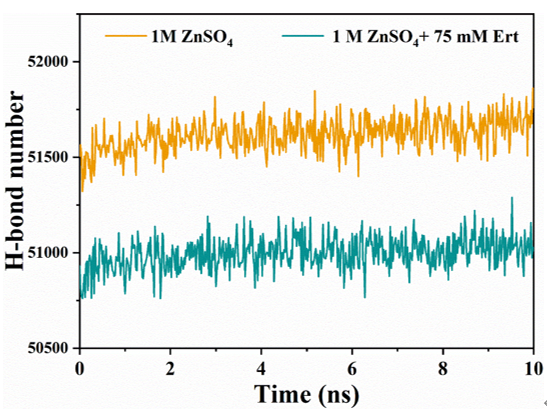


**Fig. S13** Time evolution of the number of H-bond for the ZSO and Ert-containing electrolytes.





**Fig. S14** The function of MSD vs. Time in the ZSO and Ert-75 electrolytes.





**Fig. S15** I-t curves of Zn||Zn symmetric cells before and after polarization in the ZSO electrolyte and Ert-75 electrolyte.


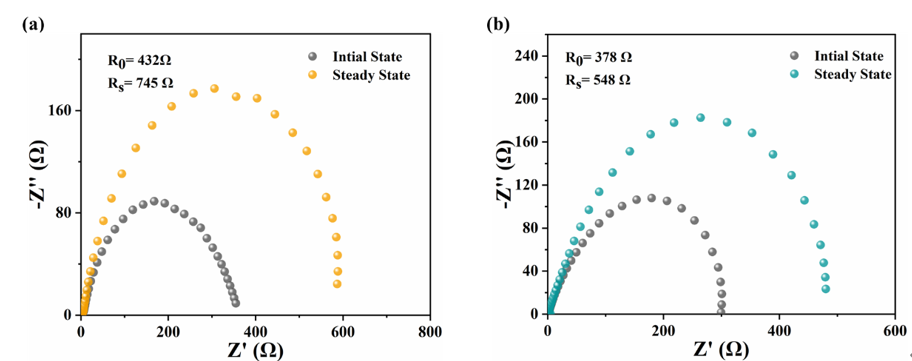


**Fig. S16** Nyquist plots of Zn||Zn symmetric cells before and after polarization in the (a) ZSO electrolyte (b) Ert-75 electrolyte.





**Fig. S17** Zn^2+^ Transfer number of Zn||Zn symmetric cells in the (a) ZSO electrolyte (b) Ert-75 electrolyte.





**Fig. S18** CV curves of Zn||Zn symmetric cells in the ZSO electrolyte.





**Fig. S19** CV curves of Zn||Zn symmetric cells in the Ert-75 electrolyte.

**

**

**Fig. S20** FT-IR spectra of Zn anode before and after immersion in various electrolytes for 12 hours.


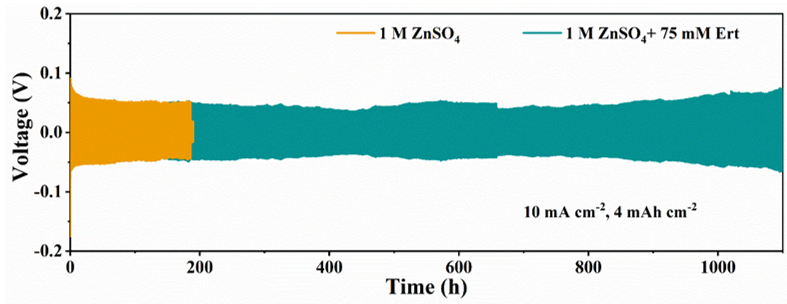


**Fig S21** GCD cycling of Zn||Zn symmetric cell at 10.0 mA cm^−2^, 4.0 mA h cm^−2^ in various electrolytes.


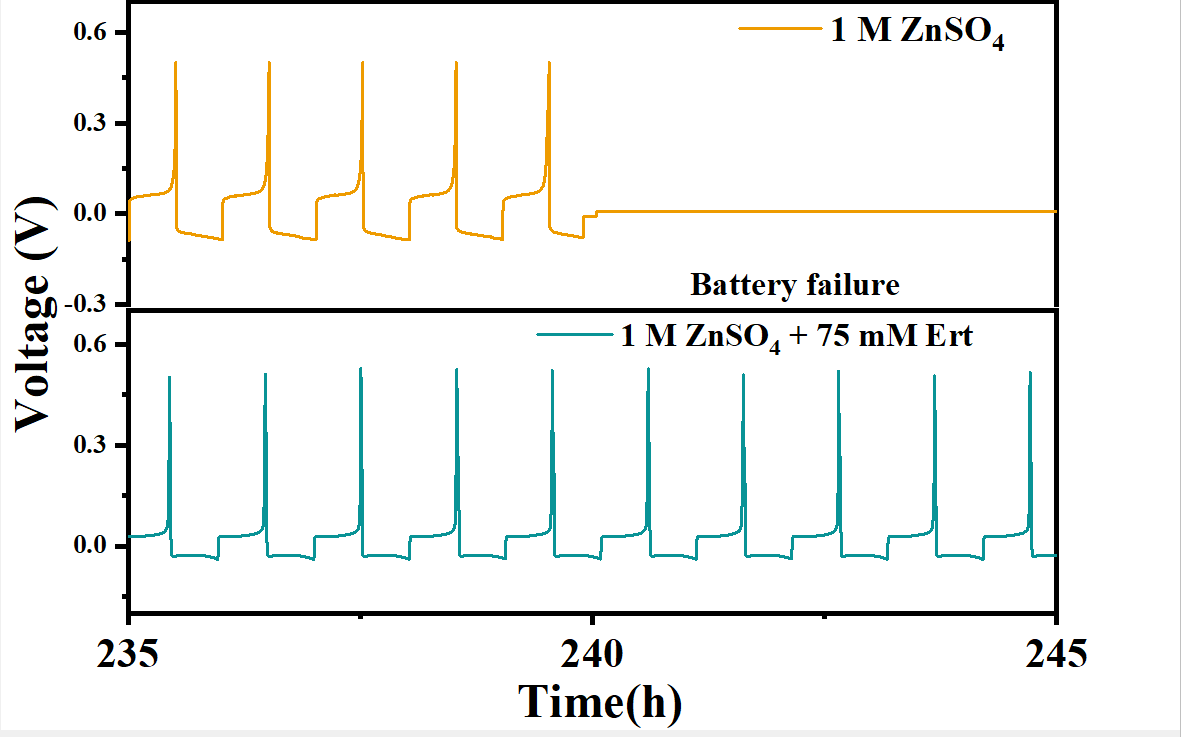


**Fig. S22** E-t curves of Zn||Cu asymmetric cell in various electrolytes (2 mA cm^-2^, 1 mA h cm^-2^)

**

**

**Fig. S23** The corresponding voltage hysteresis for Zn||Cu cells tested in the ZSO and Ert-75 electrolytes.

**
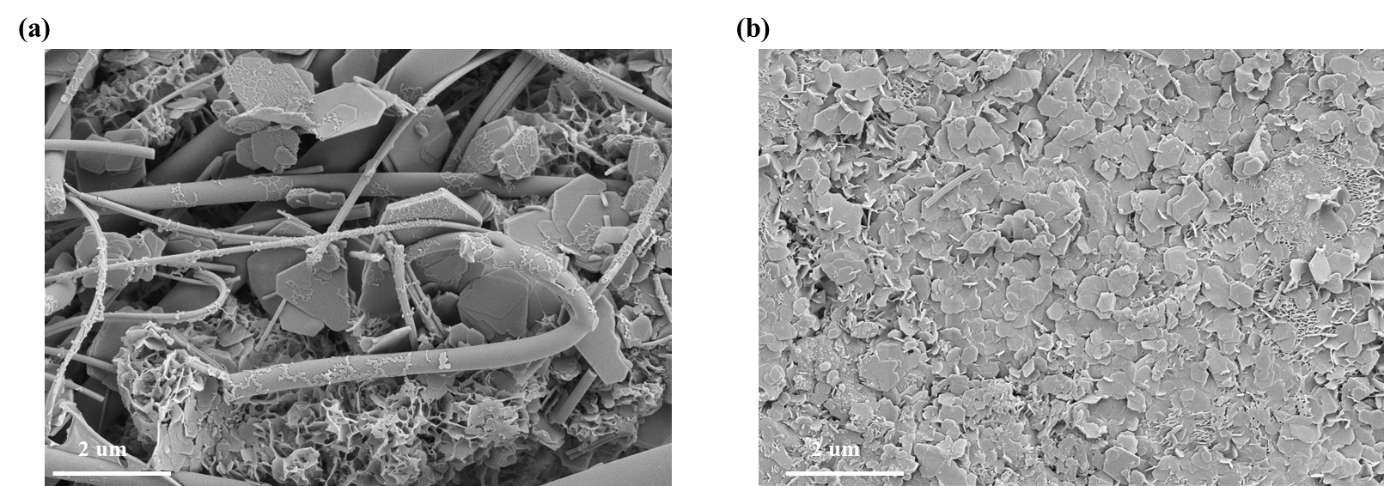
**

**Fig. S24** SEM morphologies of Zn deposition on the zinc anode in the (a) ZSO electrolyte and (b) Ert-75 electrolyte.





**Fig. S25** XRD patterns of Zn anodes after deposition in the ZSO and Ert-75 electrolytes.

**
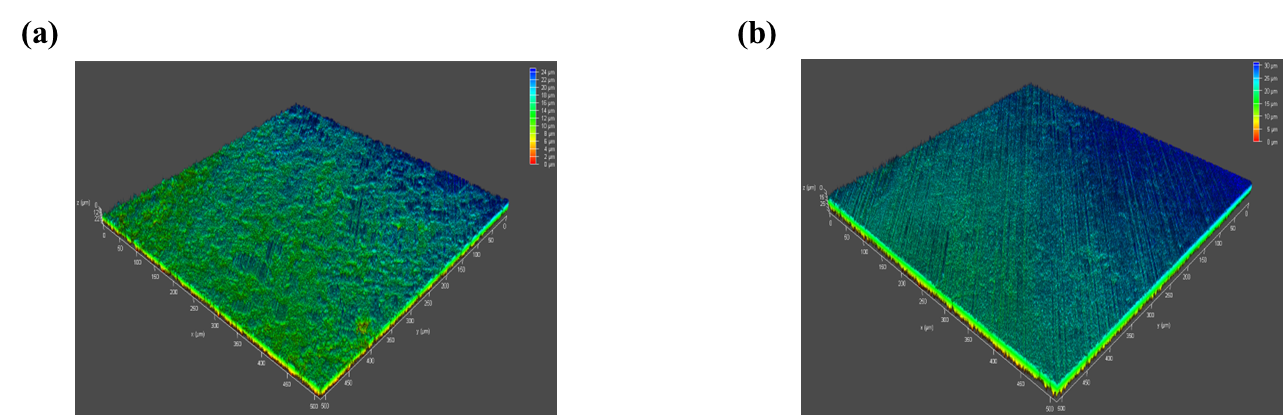
**

**Fig. S26** CLSM images of Zn anode after 50 cycles at 2 mA cm^−2^ and 1 mA h cm^−2^ in the (a) ZSO and (b) Ert-75 electrolytes.

**
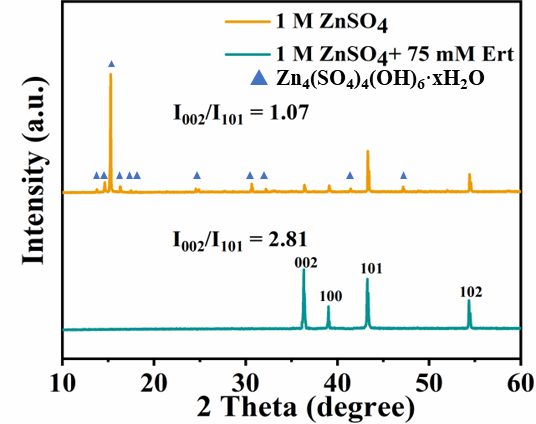
**

**Fig. S27** XRD patterns of Zn anode after 50 cycles at 2 mA cm^-2^ and 1 mA h cm^-2^.





**Fig. S28** XRD patterns of Zn anode immersed in the ZSO and Ert-75 electrolytes **for 7 days.**


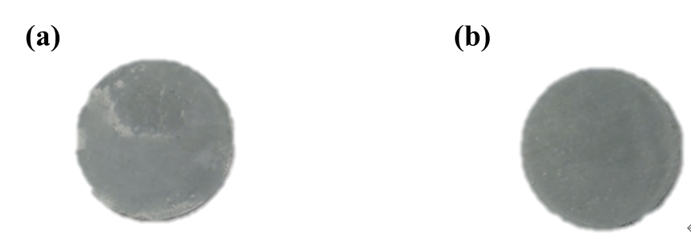


**Fig. S29** Optical photograph of Zn anodes tested in the (a) ZSO and (b) Ert-75 electrolytes after cycling at 2 mA cm^-2^ and 1 mA h cm^-2^ for 50 cycles, respectively.

**
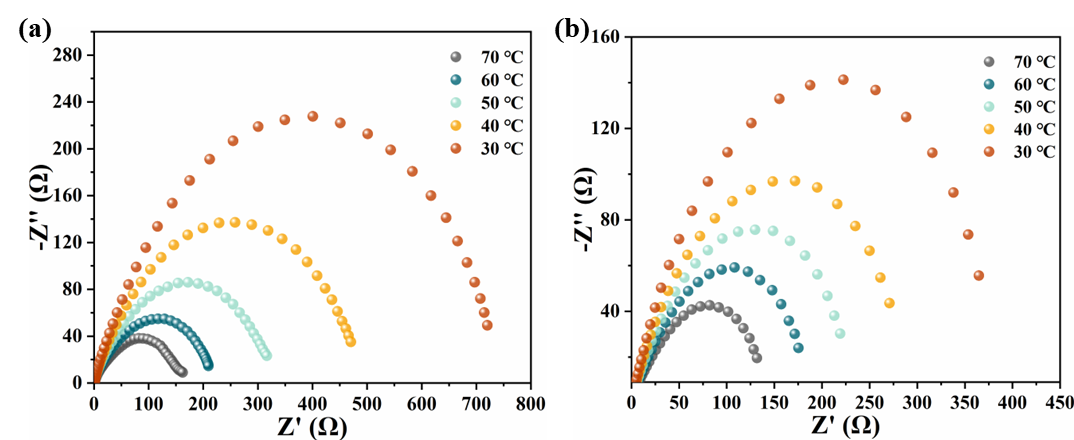
**

**Fig. S30** EIS curves of the Zn‖Zn symmetric cell in the (a) ZSO and (b) Ert-75 electrolytes under different temperatures.


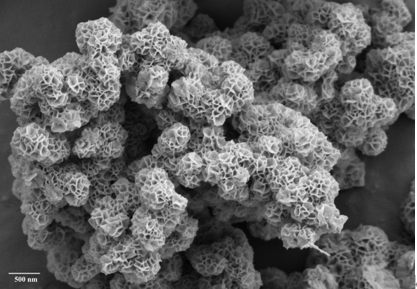


**Fig. S31** SEM image of as-prepared δ-MnO_2_ cathode.





**Fig. S32** XRD pattern of as-prepared δ-MnO_2_ cathodes.

**
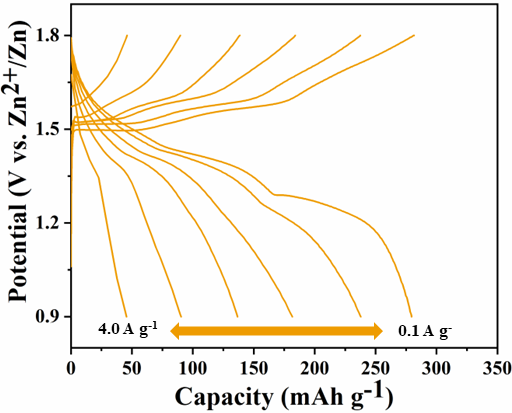
**

**Fig. S33** GCD curves of full cells in the ZSO electrolyte.


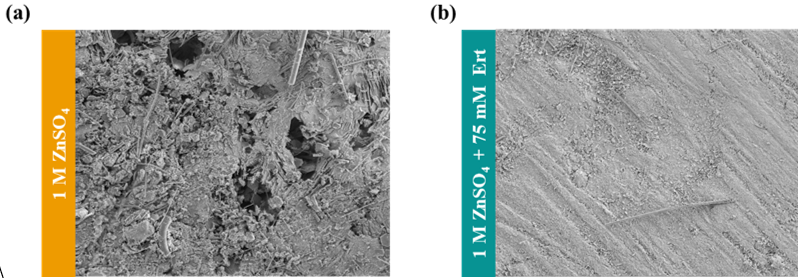


**Fig. S34** SEM images of Zn anodes after 100 cycles at 1.0 A g^−1^ the ZSO and Ert-75 electrolytes.

**

**

**Fig. S35** Self-discharge curves in the ZSO (top) and Ert-75 electrolytes (bottom).





**Fig. S36** EIS curves of full battery in the ZSO and Ert-75 electrolyte.

**Reference:**

[1] H. Luo, J. Jiang, M. Li, K. Sun, Y. Zheng, *J. Colloid Inteface Sci.* **2024**, *654*, 289.

[2] J. Cao, D. Zhang, R. Chanajaree, Y. Yue, X. Zhang, X. Yang, C. Cheng, S. Li, J. Qin, J. Zhou, Z. Zeng, *ACS Appl. Mater. Interfaces* **2023**, *15*, 45045.

[3] J. Li, Z. Guo, J. Wu, Z. Zheng, Z. Yu, F. She, L. Lai, H. Li, Y. Chen, L. Wei, *Adv. Energy Mater.* **2023**, *13*, 2301743.

[4] X. Shen, R. Zhang, P. Shi, X. Chen, Q. Zhang, *Adv. Energy Mater.* **2021**, *11*, 2003416.
